# Role of mitogens in normal and pathological liver regeneration

**DOI:** 10.1097/HC9.0000000000000692

**Published:** 2025-04-30

**Authors:** Massoud Vosough, Bahare Shokouhian, Mohammad Amin Sharbaf, Roya Solhi, Zahra Heidari, Homeyra Seydi, Moustapha Hassan, Ezhilarasan Devaraj, Mustapha Najimi

**Affiliations:** 1 Department of Regenerative Medicine, Cell Science Research Center, Royan Institute for Stem Cell Biology and Technology, ACECR, Tehran, Iran; 2 Experimental Cancer Medicine, Institution for Laboratory Medicine, Karolinska Institutet, Stockholm, Sweden; 3 Department of Pharmacology, Hepatology and Molecular Medicine Lab, Saveetha Dental College, Saveetha Institute of Medical and Technical Sciences, Chennai, India; 4 Laboratory of Pediatric Hepatology and Cell Therapy, Institute of Experimental and Clinical Research (IREC), UCLouvain, Brussels, Belgium

**Keywords:** hepatectomy, liver mitogens, liver neoplasm, normal/pathological liver regeneration, signaling

## Abstract

The liver has a unique ability to regenerate to meet the body’s metabolic needs, even following acute or chronic injuries. The cellular and molecular mechanisms underlying normal liver regeneration have been well investigated to improve organ transplantation outcomes. Once liver regeneration is impaired, pathological regeneration occurs, and the underlying cellular and molecular mechanisms require further investigations. Nevertheless, a plethora of cytokines and growth factor-mediated pathways have been reported to modulate physiological and pathological liver regeneration. Regenerative mitogens play an essential role in hepatocyte proliferation. Accelerator mitogens in synergism with regenerative ones promote liver regeneration following hepatectomy. Finally, terminator mitogens restore the proliferating status of hepatocytes to a differentiated and quiescent state upon completion of regeneration. Chronic loss of hepatocytes, which can manifest in chronic liver disorders of any etiology, often has undesired structural consequences, including fibrosis, cirrhosis, and liver neoplasia due to the unregulated proliferation of remaining hepatocytes. In fact, any impairment in the physiological function of the terminator mitogens results in the progression of pathological liver regeneration. In the current review, we intend to highlight the updated cellular and molecular mechanisms involved in liver regeneration and discuss the impairments in central regulating mechanisms responsible for pathological liver regeneration.

## INTRODUCTION

Liver is the primary organ for metabolism, biosynthesis of proteins and other bioactive molecules, detoxification, xenobiotic metabolism, and bile secretion. It is also the second largest solid organ in the human body, composed of the right and left lobes. At the tissue level, the liver is subdivided into several hepatic lobules containing several cell types well organized between a central vein and the portal triad. The liver cell types display different functions and embryonal origins, including hepatocytes, stellate cells, KCs, cholangiocytes, and endothelial sinusoidal cells. Each liver cell can be affected by various etiologies, including infection, inflammatory factors, chemicals, and carcinogens, which cause different liver disorders and lead to critical health concerns.^1^^,^^2^ However, the liver can restore its initial mass thanks to its capacity to regenerate the tissue lost post-injury.^3^

Depending on whether the loss of tissue is acute, typically observed after partial hepatectomy or mild injuries, or chronic, which generally happens in chronic liver diseases, different cellular and molecular mechanisms are activated to repair, compensate, or restore missing mass. The present review discusses cellular and molecular mechanisms governing liver regeneration and highlights the role of the most important players in promoting cell proliferation, known as “mitogens.”

## UNIQUE CAPACITY OF LIVER REGENERATION, ADVANTAGES, AND THREATS

The high regeneration potential of the liver was studied first by Higgins et al in 1931. They proposed a classic model to study hepatic regeneration in rats, where 2 out of 4 liver lobes are removed. This model has been refined and used many times to understand liver regeneration mechanisms and to improve outcomes in patients undergoing extensive liver resection for liver cancer or other underlying liver diseases.^4^^,^^5^ Indeed, from those studies, it has been clearly shown that the damaged liver can properly adjust its primary size to adapt to the body’s needs, a phenomenon called “hepatostat.”^6^

While a few studies have suggested that liver progenitor cells (LPCs) may contribute to liver regeneration, accumulating evidence indicates that their role is minimal following partial hepatectomy, with mature hepatocyte proliferation being the predominant mechanism of liver regeneration.^7^^–^^9^ Hepatocytes and biliary duct epithelial cells can dedifferentiate to LPCs in the context of liver regeneration,^10^^–^^12^ and similar to the embryonic state, undergo the differentiation procedure once again. Going through the differentiation process can increase the risk of developing liver cancer due to the similarities among the activated signaling pathways involved in hepatogenesis and hepatocarcinogenesis. Repeated irritation in chronic conditions during pathological liver regeneration can raise the risk of cancer even further.^13^ Several genes, signaling pathways, and proteins are involved in normal and pathological liver regeneration, which are the main focus of this article.

## PHYSIOLOGICAL LIVER REGENERATION

Physiological liver regeneration occurs consequently through 2 major patterns: acute liver injury caused by xenobiotics and post-resection liver injury. Acute liver injury is defined as an acute attenuation of the liver functions due to infection, chemicals/toxins, alcohol, and flare of auto-immune diseases, and is associated with high morbidity and mortality rates.^14^ This injury usually occurs when the extent of the damaged tissues exceeds the liver’s regenerative capacity and is mainly associated with the massive death of hepatocytes, especially in the centrilobular area (intoxication etiologies).^15^ and diffuse liver involvement (in viral infections) in the context of KC infiltration.^16^ Necrosis, apoptosis, or necro-apoptosis may occur following acute liver failure.^17^ However, following the hepatic injury, remaining healthy mature hepatocytes will regenerate the liver tissue, while activated LPCs could play a role in extended hepatic injuries with more than 50% involvement of hepatocytes.^18^ After ALF, the primary phase of liver regeneration is the promotion of hepatocyte mitosis, which is supported by several intrahepatic and extrahepatic pathways activation.^19^^,^^20^ After hepatic resection, liver regeneration mainly relies on the mitosis of the remaining hepatocytes, without any involvement of the activated LPCs.^21^

## MOLECULAR MECHANISMS OF NORMAL LIVER REGENERATION

Physiological liver regeneration occurs immediately after hepatic injury.^22^ In normal physiological conditions, hepatic cells are in the G_0_ state of the cell cycle. In response to acute liver injury or post-hepatectomy, those cells switch rapidly to the G_1_ phase thanks to different growth factors and cytokines called “mitogens”.^23^^,^^24^ At the early stage of liver regeneration, the liver mass is compensated by the remnant hepatocytes proliferation.^25^ In addition, the increased ratio of blood flow to the liver mass will induce a shear stress on the sinusoidal endothelial cells (SECs), leading to a change in their phenotype by Klf-2,^26^^,^^27^ and an activation of the Wnt pathway. In addition, low PO_2_ of the portal vein can trigger liver regeneration.^28^ In physiological liver regeneration, each hepatocyte can renew itself through simple mitosis induced by intracellular and extracellular inductions caused by mitogens.^29^

**FIGURE 1:**
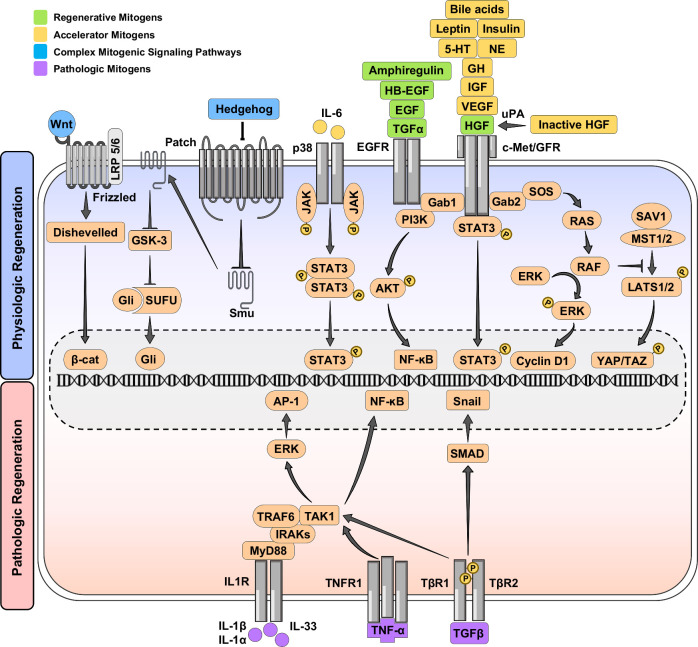
Signaling pathways involved in physiological and pathological liver regeneration. The upper part illustrates 3 types of mitogens promoting normal liver regeneration, including regenerative (green), accelerator (yellow), and complex (blue) mitogens, and the lower part indicates mitogens (purple) stimulating pathological liver regeneration.

Regenerative mitogens are critical for liver regeneration, including HGF and EGF receptor ligands, including EGF, transforming growth factor-α (TGF-α), heparin-binding EGF-like growth factor (HB-EGF), and amphiregulin. Furthermore, bile acids (BAs), norepinephrine (NE), serotonin, VEGF, insulin, insulin-like growth factor-1 (IGF1), growth hormone (GH), leptin, interleukin-6 (IL-6), and tumor necrosis factor-α (TNF-α) are accelerator mitogens. Moreover, complex pathways of mitogens, including Wnt/β-catenin and hedgehog pathways, are involved in liver regeneration.^28^^,^^30^ Eliminating regenerative mitogens significantly reduces hepatocyte proliferation and liver regeneration. However, accelerator mitogens and complex pathways are not essential for hepatocyte proliferation, and the ablation of these factors only delays the regeneration process^31^ (Figure 1). In the following subheadings, different mitogens will be discussed thoroughly.

### Regenerative mitogens

Regenerative mitogens are involved in the initial phase of liver regeneration after hepatectomy, in which the compensatory hypertrophy of the remaining liver tissue occurs after the activation of hepatocytes. The mitogens in this phenomenon stimulate the proliferation of hepatocytes and other liver cells, leading to the restoration of liver mass. The presence of these mitogens is necessary, and their impairment causes a significant defect or even complete interruption in the regeneration process, as manifested by the delayed activation of transcription factors.

#### Hepatocyte growth factor

HGF, firstly extracted in 1986 from rat platelets, is the most potent mitogen for hepatocytes.^32^ HGF or scatter factor is a pleiotropic factor affecting cellular growth, motility, and morphogenesis. This essential factor for hepatocyte proliferation is locally secreted by stromal cells, including KCs, endothelial cells, and HSCs. Studies have shown that HGF levels are higher in patients without liver failure, and the sera levels of VEGF, HGF, and IL-6 have been described as promising regeneration predictive markers in mice.^33^ In the injured liver, HSC, activated in response to reactive oxygen species, and damage-associated molecular patterns are the primary producers of HGF and thus play a vital role in liver regeneration.^34^ Immediately post-hepatectomy, HGF is transformed by urokinase plasminogen activator into its active form^35^ and acts through tyrosine-protein kinase Met (c-Met) receptor.^36^ In fact, HGF binding to c-Met activates growth factor receptor-bound protein 2 (Grb-2), Grb-2-associated binder-1 (Gab-1), SHC adaptor proteins, salt overly sensitive (SOS) signaling pathway, RAS, ERK/MAPK, PI3K/AKT signaling pathway, and signal transducer and activator of transcription 3 (STAT3), which induce DNA synthesis and mitosis.^37^ This signaling pathway is crucial for liver regeneration, and studies have shown that HGF-knockout mice exhibited defects in hepatocyte proliferation and delays in the healing process after acute liver injury or partial hepatectomy.^38^^,^^39^ HGF has been extensively studied in murine models, and studies have shown that HGF levels are significantly correlated with the degree of remnant liver growth before stage 2 of the associating liver partition and portal vein ligation for the staged hepatectomy (ALPPS) procedure. A preclinical study demonstrated that *HGF*-based gene therapy by in vivo electroporation might be useful after hepatic resection in cirrhotic livers by stimulating liver proliferative and collagenolytic capacities through activation of MAPK p44/p42 signaling pathway, as well as accelerating functional recovery.^40^

Besides, cohort observational studies have indicated that regardless of the type of underlying disease and the percentage of resected tissue, a significant increase in the levels of released HGF in the peripheral blood is observed immediately after hepatectomy. However, since HGF production is a non-parenchymal cell-dependent factor, embedded mainly in the extracellular matrix (ECM), the exact amount and timing of the HGF peak are related to the percentage of the resected liver.^41^

In a healthy liver, the HGF is sequestered within the ECM, which is crucial for regulating the availability of HGF for cellular signaling. Following liver injury, the rapid degradation of the ECM facilitates the release of HGF, triggering an initial wave of signaling essential for stimulating hepatocyte proliferation.^41^ The degradation of ECM is mediated by various proteolytic enzymes, including matrix metalloproteinases (MMPs), which break down ECM components and release sequestered growth factors like HGF into the adjacent tissue. The activation of HGF is further promoted by urokinase plasminogen activator, which converts inactive HGF into its active form shortly after hepatectomy. The interaction of HGF with c-Met activates several downstream signaling pathways that are crucial for cell growth, differentiation, survival, metabolism, proliferation, and anti-apoptotic mechanisms. Studies indicate that knockout models lacking HGF exhibit significant defects in hepatocyte proliferation and show delayed recovery following acute liver injury or partial hepatectomy.^35^^–^^37^

Recent studies on mechanisms related to HGF have revealed findings about its interaction with the liver microenvironment, crosstalk with other pathways, and regulation by the ECM. The liver microenvironment includes the SECs, HSCs, and KCs.^42^ The HGF/c-MET signaling axis contributes to modulating the liver’s microenvironment through various processes, including ECM remodeling, inflammation, angiogenesis, and invasion, to promote tumor progression and metastasis.^43^ Understanding the role of HGF in modulating these interactions and protecting non-parenchymal cells could lead to novel therapeutic strategies for liver diseases.^42^ Given its importance in tumor progression and therapeutic resistance, the HGF/c-MET axis has emerged as a potential target for cancer treatment.^44^ HGF/c-Met signaling also interacts with other pathways, such as JAK/STAT3, PI3K/Akt/NF-κB, and Ras/Raf, to promote cell proliferation, growth, and survival.^45^ The Hippo pathway, particularly its coactivators YAP/TAZ, is involved in both normal and abnormal liver regeneration. During normal regeneration, YAP/TAZ activation promotes bile duct network formation and interacts with other pathways to recruit KCs and activate stellate cells. However, in chronic liver disease, prolonged YAP/TAZ activation can lead to fibrosis and an increased risk of hepatocarcinoma.^46^ ECM components can either promote or inhibit c-MET activation by acting as bridging molecules or sequestering HGF.^47^ The activation of plasminogen facilitates the release and activation of ECM-bound HGF.^48^ HGF synergistically enhances myoblast migration on ECM proteins by inducing ERK phosphorylation and increasing production of MMP.^49^ Similarly, HGF enhances MMP activity in endothelial cells by upregulating MT1-MMP synthesis and activating MMP-2.^50^ These findings highlight the complex interactions among HGF, ECM, and MMPs in cancer progression, tissue regeneration, and angiogenesis.

On the clinical side, a trial (NCT00225901) has investigated the safety and efficacy of recombinant human HGF (rh-HGF) in patients with fulminant hepatitis. It could demonstrate tolerability, but further investigations are needed to determine the effectiveness of administering exogenous HGF in the clinic.^51^ The clinical trial NCT00225901 investigated the safety and efficacy of rh-HGF in patients with fulminant hepatitis. The results indicated that rh-HGF was generally well-tolerated by the participants; however, it highlighted the need for further investigation into its efficacy as a therapeutic agent for promoting liver regeneration. The trial NCT02083768 focuses on the safety and initial efficacy of HGF gene therapy in patients diagnosed with acute liver failure. The findings could provide valuable data on the potential benefits of gene therapy as a novel treatment approach for this condition. The clinical trial NCT03017016 evaluates the safety and efficacy of the HGF gene therapy drug HGF-ung1 specifically in patients with acute liver failure. This study employs a targeted gene therapy strategy aimed at enhancing the liver’s regenerative potential by increasing the concentration of HGF at the injury site. The outcomes are critical in determining whether HGF-ung1 can effectively promote hepatocyte proliferation and improve overall liver recovery. The current trial, NCT03924318, investigates another variant of HGF gene therapy, known as HGF-PE. This trial focuses on assessing the safety and therapeutic potential of HGF-PE in patients with liver conditions requiring regeneration. The results may provide insights into how variations in gene therapy can influence treatment outcomes and enhance liver recovery.

#### Epidermal growth factor receptor ligands

The epidermal growth factor receptor (EGFR) is a member of the ErbB family that interacts with other ErbB receptors and regulates cell proliferation and growth.^52^ Among 7 possible ligands of EGFR,^53^ 4 of them, including TGF-α, EGF, HB-EGF, and amphiregulin, have a critical role in liver regeneration.^54^ By binding to EGFR, the mentioned ligands will activate several intracellular pathways, including the RAS/RAF, PI3K/Akt, phospholipase Cγ, STAT3, and Src kinase pathways, involved in the regulation of cell proliferation and growth, indicating the critical role of EGFR in the initial phases of liver regeneration.^55^

Considerable evidence has emerged on the importance of EGFR signaling pathways in liver inflammatory responses to injuries. Preclinical studies reported highly induced levels of EGFR in hepatocytes alongside high levels of HB-EGF, TGF-α, EGF, and amphiregulin after partial hepatectomy. This resulted from an upregulation in disintegrin and metalloproteinases-17 (ADAM-17), essential factors for the shedding of ErbB ligands.^56^ The proliferative and survival signals of EGFR ligands were demonstrated in vivo after intraperitoneal injection of these factors.^57^^,^^58^ Natarajan and colleagues indicated that ErbB1 knocked-down mice showed higher mortality rates and higher serum transaminase levels, implying liver injury. Reduction in cyclin D1, *cdk2*, and *cdk1* expression delayed cell cycle progression in the G_1_–S phase and consequently decreased hepatocyte proliferation. Inhibited liver regeneration resulted in increased blood TNF-α as a compensatory mechanism and extended c-Jun activation.^59^ In another study by Paranjpe et al,^60^ silencing the *EGFR* mRNA was demonstrated to be associated with a significant decrease in liver regeneration, leading to hepatic decompensation.

Although EGFR ligands have been extensively studied in animal models, there is limited information on their application in clinical studies, whether they induce or accelerate liver regeneration post-hepatectomy. Considering the high rate of mortality post-hepatectomy (liver resection), more research studies regarding the application of this mitogen in boosting the compensation of lost tissues are necessary.

### Accelerator mitogens

Accelerator mitogens play a critical role in the liver tissue’s remodeling and restoring liver function, modulating the early phase of liver regeneration, and controlling the timing of essential transcription factors associated with hepatocyte proliferation. Accelerator mitogens are extracellular signals that interact and synergize with regenerative mitogens to promote liver regeneration after hepatectomy. Deprivation of accelerator mitogens would delay but not abrogate liver regeneration after partial hepatectomy.

#### Bile acids

BAs, mainly consisting of cholesterol, are essential for various physiological functions, such as fat digestion, cholesterol metabolism, vitamin absorption, and immune system homeostasis.^61^ Previous studies showed that BAs are crucial metabolic compounds that promote liver regeneration through different signaling pathways. In fact, urinary BA output increases, and a fetal-type molecular species of BA reappears in the urine on day 3 and day 7 after hepatectomy. BA salts can interfere with G protein-coupled BA receptor-1 (TGR5) and farnesoid X receptor (FXR).^62^ TGR5 is a cell surface receptor known for regulating BAs homeostasis and anti-inflammatory responses through interference with KC functions.^63^ FXR is classified as a nuclear receptor that binds to DNA and regulates the gene expression related to the liver metabolic activity.^64^ FXR is expressed in the liver and terminal ileum^65^ and plays a crucial role in sustaining intrahepatic BA levels at safe concentrations and preventing BA overload’s toxic consequences. The synthesis of BAs occurs essentially by cholesterol-7α-hydroxylase (CYP7A1) and requires FXR expression in the ileum and the liver.^65^

After the activation of TGR5 and FXR by BAs, the quiescent hepatocytes enter the cell cycle. FXR regulates the cell cycle progression through Foxm1b, a critical factor for G1/S and G2/M progression, and the FXR/FGF19/FGFR4 signaling axis.^66^^,^^67^ In addition, the FXR regulates the BA concentration and prevents the apoptosis and necrosis of hepatocytes through the FGF19/FGFR4/βKlotho pathway.^68^ In fact, FXR’s knockdown in mice causes impairment of liver regeneration.^69^ Also, lack of TGR5 in mice is associated with high levels of BAs, severe inflammatory responses, and delayed liver regeneration.^70^ Although BAs play a crucial role in the early stages of liver regeneration, BA overload could ultimately result in liver toxicity and delayed liver regeneration following liver resection.^71^

#### Norepinephrine

Recent studies have highlighted the mechanisms by which NE influences liver regeneration. NE is released during sympathetic nervous system activation, and its interactions with adrenergic receptors enhance hepatocyte proliferation following liver injury. It has been shown that NE induces cell cycle and DNA synthesis through its α-adrenergic receptors^72^ and improves liver regeneration after the antagonization of the TGF-β inhibitory effect on hepatocytes.^73^ HSCs express adrenoceptors, produce catecholamines, and are growth-inhibited by adrenoceptor antagonists.^74^^,^^75^ Mice lacking NE showed reduced HSC activation and fibrogenic responses, which can be rescued by adrenoceptor agonists.^75^ Conversely, sympathetic nervous system inhibitors enhance oval cell accumulation and may improve liver repair.^74^ These findings suggest that the sympathetic nervous system regulates liver repair by modulating HSC and oval cell phenotypes, potentially offering novel therapeutic approaches for liver damage and fibrosis. The β2-adrenergic receptor is also involved in liver regeneration; its deletion leads to increased mortality and impaired liver function post-hepatectomy. β2-adrenergic receptor promotes liver regeneration through crosstalk with c-met and the activation of ERK signaling.^76^ Furthermore, NE may work synergistically with other mitogens, like HGF and EGF, to amplify the regenerative response. NE enhances the mitogenic effects of EGF in the liver by interfering with α-adrenergic G protein-coupled receptors and improving hepatocyte proliferation.^77^ Alongside the abovementioned effects, NE can increase the production of EGF and HGF by Brunner glands, the 2-key regenerative mitogens involved in liver regeneration.^78^ Moreover, during the perioperative period of liver transplantation, NE is often administered to address hemodynamic instability and improve organ perfusion and oxygen supply. However, a retrospective cohort study of 430 children investigated the impact of NE infusion on the prognosis of pediatric living donor liver transplantation. This study indicated that intraoperative NE administration was related to poor prognosis, suggesting that NE might not be the best choice for circulatory support during pediatric living donor liver transplantation.^79^

#### Serotonin

Serotonin, or 5-hydroxytryptamine (5-HT), is a critical hormone of the central nervous system that is produced by the enterochromaffin cells of the gastrointestinal tract. It is deeply involved in many physiologic functions, including gastrointestinal motility, cell proliferation, apoptosis, and platelet aggregation, as well as liver functions such as hepatic energy metabolism, hepatic fibrosis, steatosis, and liver regeneration.^80^ Furthermore, the balance between liver fibrogenesis and regeneration in chronic liver injuries appears to be primarily controlled by serotonin signaling. Serotonin is commonly carried by platelets and has been shown to promote liver regeneration in mice after injury through serotonin receptors (HTr2A and HTr2B). In thrombocytopenic mice, a serotonin agonist improved liver cell proliferation.^81^ In addition, the expression of 5-HT2A and 2B serotonin receptors significantly increased after hepatectomy, while antagonists of 5-HT2A and 2B receptors were able to inhibit liver regeneration.^82^ A notable study in a murine PHx model indicated the role of 5-HT in the 5-HT–pERK–YAP axis in liver regeneration.^83^ Moreover, selective serotonin reuptake inhibitors and serotonin noradrenaline reuptake inhibitors reduced the intraplatelet 5-HT concentration. A clinical study showed that perioperative selective serotonin reuptake inhibitor/serotonin noradrenaline reuptake inhibitor intake was significantly associated with a high incidence of morbidity and postoperative hepatic dysfunction.^84^

#### Vascular endothelial growth factor

VEGF is the major angiogenesis regulator and induces the transition from G_0_ to G_1_ cell cycle phase.^85^ VEGF operates via at least 3 receptors, VEGFR-1, VEGFR-2, and VEGFR-3.^86^ VEGF expression was reported to increase significantly after partial hepatectomy, and was associated with the high proliferation of SECs and hepatocytes.^87^^,^^88^ In the coculture of hepatocytes with SECs, VEGF enhanced the proliferation of hepatocytes and HGF expression through VEGFR-1.^89^ Furthermore, expression of VEGFR-2, the primary regulator of the VEGF performance, increased during liver regeneration and enhanced the VEGF activity through the regeneration process.^90^

#### Insulin

It has been suggested that insulin has promising roles in liver growth and regeneration. Indeed, liver regeneration implies mitochondrial metabolic activities, strongly associated with insulin to preserve a high ketone-body ratio.^91^ After partial hepatectomy, the uptake of insulin and glucagon is increased by hepatocytes, which induces liver regeneration.^92^^,^^93^ The increased endogenous insulin levels reported after 48 hours post-hepatectomy suggest the insulin heterotrophic function at the early stages of liver regeneration.^94^ In addition, studies on the human population showed the positive effects of intraportal insulin infusion on liver graft regeneration after transplantation.^95^

#### Insulin-like growth factor

Insulin-like growth factors (IGFs) are produced in the liver in response to GH and are critical for the proliferation and differentiation of hepatocytes.^96^ IGFs act through the IGF receptor (IGF1R) and are modulated by at least 6 different IGF binding proteins (IGFBPs), which have a greater affinity to IGF compared to IGF1R and play a critical role in regulating IGF signaling.^97^ These factors are associated with insulin functionality; however, they display a higher growth-induction capacity.^98^ It has been shown that IGF1 stimulates the cell cycle by DNA synthesis and promotes HGF production by non-parenchymal liver cells such as HSCs.^99^^,^^100^ In the context of IGFBPs, IGFBP-1 is reported to be highly expressed in liver regeneration, while its deficiency is associated with abnormal liver regeneration.^101^ Moreover, an increase in IGFBP-4 concentrations was seen 12–24 hours after a partial hepatectomy, which might be related to the modulation of IGF expression during liver regeneration.^102^ Importantly, IGF signaling is different in normal and pathological liver regeneration. While IGF1 plays a more influential role in normal liver regeneration, IGF2 is more critical in regeneration after chronic liver injury. IGF1 induces cellular senescence, improves NASH, and reduces fibrosis and cirrhosis. However, animal studies revealed that IGF2, expressed by pericentral hepatocytes, enhances hepatocyte proliferation after pathological and chronic liver injuries.^103^^–^^105^

#### Growth hormone

GH is involved in the modulation of liver growth directly via its receptor and indirectly through IGF1 enhancement and IGF1R activation.^106^ It was shown that GH could adjust EGFR expression and enhance EGFR tyrosine phosphorylation, an essential event in liver regeneration.^107^^,^^108^ Blocking GH agonists has been reported to be related to a decrease in liver regeneration capacity and survival rate of the rats following partial hepatectomy.^109^ In another study in 2021, it was shown that GH eliminates the excessive immune response observed after a partial hepatectomy through the induction of H2-Bl/HLA-G, which is associated with liver regeneration and improved survival rate.^110^ A notable systematic study summarized current evidence on the cytokine-mediated and growth factor-mediated signaling pathways in liver regeneration for the benefit of clinicians and discussed their suitability for individual mediator-based regeneration predictions in patients. The study reported that the GH–IGF1–IGF1R axis is necessary for liver regeneration after partial hepatectomy in liver-specific IGFIR knockout mice.^103^ Besides, GH therapy has been shown to improve liver regeneration and protein synthesis after hepatectomy in patients with cirrhosis and HCC (NCT03420144 and NCT05253287).

#### Leptin

Leptin is mainly produced by adipose tissue and small intestine enterocytes, and it is a hormone that regulates liver functions, including glucose and lipid metabolism, and plays a protective role against fat accumulation and steatosis.^111^^–^^113^ Several studies have investigated its role in liver regeneration.^114^ Many receptors are associated with leptin functions, including LEPR-a, LEPR-b, LEPR-c, LEPR-d, LEPR-e, and LEPR-f.^115^ However, LEPR-b has a more prominent role in transducing different signals to the hepatic cells, including JAK-2/STAT3, IRS/PI3K, and SHP-2/MAPK.^116^ In addition, a lack of leptin can decrease the EGFR and indirectly induce liver regeneration.^117^ Leptin-deficient mice had impaired liver regeneration post-hepatectomy or toxin damage compared to the control groups.^118^ Furthermore, lack of leptin can affect DNA synthesis, inhibit mitosis, and change the hepatocyte phenotype to a non-proliferative state.^119^

#### Tumor necrosis factor-alpha

It was revealed that TNF-α has protective effects during liver regeneration.^120^ This factor is produced by macrophages and acts via 2 main receptors: TNFR-1 and TNFR-2.^121^ In normal conditions, TNF-α induces hepatocyte apoptosis and liver failure. However, there are paradoxical reports about the TNF-α, and it seems that its effects on the liver depend on NF­κB activation.^122^ Indeed, if the expression of TNF-α is associated with NF­κB activation, this will result in DNA synthesis and hepatocyte proliferation.^123^ Same as EGF and HGF, TNF-α expression is associated with Akt phosphorylation, and TNF has a promoting role in the mitogenic effect of EGF and HGF.^124^ Furthermore, the lack of TNF-α expression is associated with lower levels of IL-6,^125^ which is critical for activating STAT3.^126^ Seemingly, TNF-α is involved in the early stages of liver regeneration; complete knockout of TNF-α did not delay regeneration, suggesting that TNF-α is not involved in the later stages of regeneration.^103^ On the other hand, another study investigated the effects of pentoxifylline on liver regeneration in 101 patients undergoing major liver resection. The study found that pentoxifylline improved liver regeneration through downregulation of TNF-α synthesis and *TGFB1* expression.^127^

#### Interleukin-6

IL-6 is an inflammatory cytokine that is highly expressed at the cell surface of hepatocytes, plays a role in different pathophysiological states,^128^ and triggers intracellular signaling pathways through its receptor (IL-6R).^129^ IL-6 is a potent hepatocyte mitogen, and the levels of IL-6 rapidly increase after hepatectomy, alongside the upregulation of TNF-α in the liver’s central vein.^130^ It was reported that IL-6 knockout mice have impaired liver regeneration,^131^ while elevated expression of IL-6 or its receptor promotes liver regeneration and hepatocyte proliferation. Although IL-6 acts as a double-edged sword, prolonged activation of the IL-6 signaling pathway during chronic liver diseases damages the liver and could ultimately result in liver carcinogenesis, as in HCC patients, elevated serum levels of IL-6 and the soluble IL-6R have been detected.^132^

### Terminator mitogens

During the final phase of liver regeneration, certain mitogens terminate the rapid proliferation of hepatocytes to avoid overgrowth and provide an adequate mass for compensation. This stage, known as the termination phase, is marked by the transition of actively proliferative hepatocytes to a differentiated and functional state once the regeneration process is complete.^133^ The precise regulation of terminator mitogens is the key difference between physiological and pathological liver regeneration. Disturbance in terminating mitogens can lead to pathological conditions in 2 ways: the lack of these signaling molecules can lead to excessive uncontrolled mitosis and development of pathologic regenerative nodules, or their long-term expression may result in chronic inflammatory conditions and fibrosis.^134^

#### Transforming growth factor-beta 1

Transforming growth factor-beta 1 (TGF-β1) is the most essential hepatocyte proliferation inhibitor, which terminates cell proliferation during hepatic regeneration. It is primarily secreted by non-parenchymal cells, including HSCs, KCs, and platelets. This factor is produced by proliferating hepatocytes. Four hours following partial hepatectomy, the expression of TGF-β1 in regenerating hepatocytes increases and peaks at 72 hours.^135^ However, during liver regeneration, the responsiveness to TGF-β1 decreases temporarily, which is essential for proceeding with hepatocyte proliferation. This transient resistance may occur due to the downregulation of TGF-β1 receptors or the formation of co-regulatory axes like SnoN–Smad2/3 and YAP-1–Smad2 complexes.^136^ Based on the literature, there are several mechanisms through which TGF-β1 can inhibit hepatocyte proliferation, and these multifaceted approaches enable TGF-β1 to play a central role in regulating liver regeneration. TGF-β1 hinders the secretion of HGF, the main regenerative mitogen after partial hepatectomy, and inhibits DNA synthesis induced by HGF, EGF, HB-EGF, and TNF-α in cultured hepatocytes. Another mechanism in this regard is TGF-β‘s regulation of the TGF-β/SMAD signaling pathway. Reduced TGF-β/SMAD signaling accelerates hepatocyte proliferation.^137^ The activation of TGF-β is necessary for inhibiting cell proliferation, and thrombospondin-1 (TSP-1) is required for TGF-β activation. Mice with TSP-1 deficiency following partial hepatectomy showed significantly reduced TGF-β/Smad signaling and accelerated hepatocyte proliferation. TSP-1 is prominently produced in liver endothelial cells in response to partial hepatectomy, and thereafter, TGF-β signaling is inhibited in the early regeneration period by upregulation of TGF-β inhibitory proteins SnoN and Ski and a downregulation of SMAD2 and SMAD4 levels, which are significantly enhanced following partial hepatectomy. These findings suggest that TSP-1 plays a crucial role in liver regeneration by regulating TGF-β signaling.^138^^,^^139^

#### Activin

Activins, as a member of the TGF-β superfamily, play an influential role in terminating liver regeneration following partial hepatectomy. Activin A level increases 12 hours after partial hepatectomy and is considered an apoptogen that induces hepatocyte growth arrest in vitro and in vivo. Like TGF-β, activin A signaling is reduced during liver regeneration because its cellular receptor levels are downregulated. However, receptor levels are restored once liver regeneration is completed.^30^ Treatment of mice lacking the type II TGF-β receptor with follistatin, an activin A antagonist, enhanced DNA synthesis and prolonged hepatocyte proliferation. Activin A also significantly affects the production of fibronectin, an essential component of the ECM required for liver regeneration. On the other hand, chronic liver injury leads to the long-term activation of autocrine TGF-β and activin A signaling, contributing to the constant status of partial epithelial–mesenchymal transition in hepatic progenitor cells. Furthermore, in hepatocytes, TGF-β and activin A signaling are key players in liver fibrosis by inducing autonomous synthesis of connective tissue growth factor (CTGF/CCN2). This partial epithelial–mesenchymal transition state and excessive ECM production may facilitate liver fibrosis, promoting the hepatic progenitor cells to participate in hepatocarcinogenesis.^140^^,^^141^

#### Bone morphogenetic proteins

Bone morphogenetic proteins (BMPs) are the other members of the TGF-β superfamily, but the signaling pathway mediated by BMPs is relatively complex. Different subunits of BMPs exhibit different or even controversial effects on hepatocyte proliferation. It seems that BMP-4 and BMP-9 inhibit the proliferation of hepatocytes; however, BMP-7 promotes hepatocyte proliferation.^142^ It has been found that BMP-4 is mainly expressed in the peribiliary stroma and endothelial cells of the liver, and its expression is decreased after hepatectomy. BMP-4 also contributes to hepatoblast differentiation in vivo through the BMP4/Smad1 axis.^142^^,^^143^ BMP-9 is constitutively expressed in healthy liver, predominantly by quiescent and activated HSCs. It was shown that treatment with BMP-9 inhibited proliferation and epithelial–mesenchymal transition, and induced the expression of hepatic maturity genes in cultured hepatocytes. Furthermore, BMP-9 mRNA expression was temporarily decreased after acute liver injury induced by partial hepatectomy or single injections of carbon tetrachloride (CCl_4_) or lipopolysaccharides in mouse models.^144^ Conversely, BMP-7 facilitates liver regeneration by promoting hepatocyte proliferation and reducing liver damage. Notably, ALK3 (BMP receptor type 1A) is expressed in healthy livers and upregulated in regenerating livers. Interestingly, BMP7 is not expressed in the liver, and circulating BMP7 is released from the kidneys, targeting the liver to promote regeneration and acting as a pro-regenerative endogenous hormone.^145^ Moreover, there are controversial findings concerning the role of BMP-2 in liver regeneration. Some studies suggested that its expression rapidly declines after partial hepatectomy, implying that BMP-2 signaling does not support hepatocyte proliferation during the regenerative process.^142^ However, others indicated that BMP-2 restoration might assist in recovery from liver fibrosis by attenuating TGF-β1 signaling.^146^ These findings point out that BMP-2 plays a complex role in liver regeneration, contributing to the wound-healing process and liver fibrosis regression, while this signaling may not directly support hepatocyte proliferation during the regenerative phase.

Considering these controversial findings, a better understanding of the underlying mechanisms of BMPs in the progression of liver disease is critically important for improving their therapeutic effect.

### The complex network of signaling pathways in liver regeneration

Liver regeneration after partial hepatectomy involves a complex network of signaling pathways that are controlled by mitogenic growth factors, cytokines, hormones, and transcription factors. The events during liver regeneration are finely tuned in time and space, and the interplay between these signaling pathways is critical for liver regeneration. However, the exact mechanisms of how these signaling pathways interact with each other have not been fully understood yet.

#### Wnt/β-catenin pathway

Wnts are glycoproteins that induce different intracellular signaling pathways in mammals.^147^ Wnt ligands induce their frizzled receptors via 2 major pathways: (1) the “canonical pathway,” which is related to β-catenin regulation and is called the Wnt/β-catenin pathway. (2) The “non-canonical pathway,” which is called Wnt/Ca^2+^ or Wnt/planar cell polarity (PCP) pathway.^148^ The Wnt/β-catenin pathway induces proliferation and improves survival rate, which is known for its function in liver regeneration.^149^ When this pathway is inactivated, the level of β-catenin is low due to its phosphorylation and degradation by the destruction complex, including Axin, glycogen synthase kinase-3β (GSK-3β), adenomatous polyposis coli (APC), and casein kinase-1α (CK1-α).^150^ This complex is responsible for the β-catenin phosphorylation, ubiquitin targeting, and proteasomal degradation. Wnt/β-catenin activation causes the arrangement of scaffold protein disheveled (DVL), which leads to disaggregation of the destruction complex and the stabilization of β-catenin. Stabilized β-catenin molecules accumulate in the cytoplasm and translocate to the nucleus.^151^ Furthermore, β-catenin can be phosphorylated by EGFR and MET, resulting in the prevention of destruction complex formation and facilitating its translocation.^152^ The Wnt/β-catenin pathway plays a vital role in liver regeneration, liver function, and hepatic tumor development.^153^ In rat models, after 70% partial hepatectomy, overexpressed Wnt1 and nuclear β-catenin are mainly increased in remaining hepatocytes. Usually, a transient increase of β-catenin occurs within 5 minutes after hepatectomy,^154^ associated with cyclinD1 and c-myc overexpression, and results in cellular proliferation.^155^ Moreover, this pathway has crosstalk with MET and EGFR, related to HGF and EGFR ligand stimulation, and enhances complete mitogen functions.^156^ In fact, it was shown that β-catenin knockdown mice had low rates of hepatocyte proliferation and delayed liver regeneration.^157^^,^^158^

#### Hedgehog pathway

The hedgehog family regulates cell fate, including proliferation, apoptosis, and differentiation.^159^ This pathway comprises signaling molecules, including Sonic (Shh), Indian (Ihh), and Desert (Dhh) Hedgehog.^160^ The principles of the Hedgehog pathway occur in primary cilia (PC), which form from centrioles at the end of the mitosis receptor.^161^ In addition, 4 components, including Patched (Ptch) receptor, signal transducer Smoothened (Smo), Glioblastoma (Gli) family, and Hedgehog ligands, regulate the Hedgehog pathway.^162^ In the absence of hedgehog ligands, Ptch inhibits the activity of Smo, which is associated with Gli phosphorylation and degradation.^163^ However, in the context of Hedgehog ligands, adhesion to Ptch and Smo activation prevents Gli degradation, which is associated with its nuclear transcription.^164^ Hedgehog ligands are produced during liver regeneration, stimulated by factors such as EGF, TGF-β, and PDGF.^165^ Injured hepatic cells produce Sonic Hedgehog and Indian Hedgehog ligands,^166^ dramatically increasing Hedgehog signaling after partial hepatectomy. This was associated with epithelial regeneration, Hippo/Yes-associated protein 1 (Yap-1) activation, and sinusoidal cell regeneration, resulting in liver regeneration.^152^

#### Yap/TAZ pathway

The hippo signaling pathway coordinates cell proliferation during physiologic organ growth.^167^ This pathway acts through the Mammalian Ste20-like kinases (MST1/2) and large tumor suppressor kinase (LATS1/2), resulting in the inhibition of yes-associated protein (YAP) and Transcriptional coactivator with PDZ-binding motif (TAZ).^168^ Hippo signaling seems to regulate hepatocyte proliferation during liver regeneration through YAP/TAZ blockage, resulting in regeneration halts.^169^ It was also suggested that liver regeneration and cell plasticity are severely impaired after YAP/TAZ blockage.^170^ However, YAP/TAZ is not necessary for liver regeneration due to the essential roles of complete mitogens.^171^

## LIVER REGENERATION MODELS

The liver has a remarkable ability to regenerate following injury, particularly through 2 primary pathways of liver regeneration: the proliferation of mature hepatocytes and the activation of liver progenitor/stem cells (LPCs).^172^^,^^173^ The proliferation of mature hepatocytes is primarily driven by mitogenic signals from certain growth factors, including HGF and EGF. These growth factors activate specific signaling pathways essential for cell division and regeneration.^174^ LPCs possess the capability to differentiate into both hepatocytes and biliary epithelial cells, thereby offering an alternative mechanism for tissue repair in the context of chronic injury.^175^ Recent studies have elucidated the interactions between regenerative and accelerator mitogens, revealing how these pathways coordinate to promote liver regeneration. For instance, BAs have been shown to modulate these pathways, enhancing the regenerative response.^176^ Furthermore, advances in genetic engineering have led to the development of mouse models with specific gene knockouts or modifications. The lack of EGFR signaling in knockout mice resulted in increased mortality rates and elevated serum transaminase levels, indicating severe liver injury. The studies found that the reduced expression of cell cycle regulators, such as cyclin D1 and cdk2, leads to delayed cell cycle progression and decreased hepatocyte proliferation, emphasizing the substantial role of EGFR ligands (such as EGF, TGF-α, HB-EGF, and amphiregulin) in promoting liver regeneration after injury.^177^ Mouse models lacking key hedgehog signaling pathway components provided insights into the role of this pathway in regulating the balance between hepatocyte proliferation and LPC activation.^178^ Genetic modifications targeting BA receptors, such as FXR, showed that BAs are a crucial accelerator of mitogens in liver regeneration. Studies involving mice with disrupted BA signaling pathways demonstrated delayed liver regeneration following hepatectomy, indicating the importance of BAs in modulating mitogen activity and promoting hepatocyte proliferation.^179^ A study on the TGF-α knockout mouse model indicated that liver regeneration proceeded normally after a 70% hepatectomy in mice, indicating that TGF-α is not essential for liver regeneration but could be important in the progression of tumors.^180^ Conversely, mouse models with overexpression of TGF-α have provided insights into its role in promoting liver regeneration. The enhanced regenerative response was associated with elevated activation of EGFR and subsequent downstream signaling pathways that promote cell division.^181^ These models have provided insights into how alterations in mitogen signalings can affect liver regeneration outcomes, enabling the exploration of potential therapeutic targets.

### Pathological liver regeneration

#### Liver zonation in liver regeneration

Liver zonation describes the organized arrangement of hepatocytes and other liver cells within the hepatic lobule, which is divided into 3 distinct zones: periportal (zone 1), midzonal (zone 2), and pericentral (zone 3). Each zone has unique metabolic functions and responds differently to a range of stimuli, including injury and regeneration. Hepatocytes initiate the cell cycle during the regeneration process, enabling them to proliferate and repair damaged tissue. The expression of zone-specific genes plays a crucial role in facilitating the functional recovery of various metabolic regions.^182^ IGF2 is a significant zonal signal produced by pericentral hepatocytes located in zone 3 during liver regeneration. This growth factor facilitates hepatocyte proliferation and contributes to the repair of tissue following liver injury, highlighting its essential function in the regenerative process.^104^ Following liver injury, different zones activate distinct signaling pathways that influence regeneration. For instance, hepatocytes in zone 1 may respond more robustly to growth factors like HGF due to their proximity to the portal triad, where these factors are abundant.^183^ The distribution pattern of growth factors and cytokines within the liver tissue also reflects zonation. Notably, the concentration of HGF may be higher in periportal regions (zone 1), facilitating the rapid proliferation of hepatocytes in that region. Similarly, the signaling of TGF-α may differ across the zones, thereby influencing the regenerative responses of hepatocytes.^184^ The expression of IGF2 in zone 3 underscores the spatial regulation of growth factors that modulate regenerative responses throughout the liver lobule.

### Liver regeneration after acute liver injury

The liver has an exceptional ability to regenerate after the loss of liver tissue due to drug and chemical-induced injury, surgical resection, infection, or trauma.^185^ This regenerative capacity is a well-documented compensatory mechanism.^186^ The administration of a single dose of CCl_4_ in mice induces the degeneration of hepatic architecture and subsequent liver necrosis. Notably, hepatocellular proliferation along with sinusoidal remodeling around necrosis initiates within 24 hours, peaks at 48 hours, and concludes by 72 hours, as reported by Michalopoulos and DeFrances.^187^ This observation underscores the liver’s inherent compensatory regeneration potential following acute chemically-induced liver injury, which is often reversible up to a certain extent. In a study, mice treated with 300 mg of acetaminophen exhibited significant liver injury followed by robust regeneration. However, when the acetaminophen dose was increased to 600 mg, liver regeneration was inhibited, resulting in sustained liver injury, reduced survival rate, cell cycle arrest, and decreased expression of cyclin D1.^188^ This highlights that when a threshold of injury is exceeded, liver regeneration could be inhibited.^188^ Therefore, the extent of liver regeneration is crucial in determining the final outcome of toxicant-induced liver injury.^188^ Timely stimulation of liver regeneration can also enhance the liver’s regenerative potential and improve survival. Conversely, inhibiting liver regeneration, such as with colchicine, an antimitotic agent, exacerbates injury and can lead to death.^186^ Mechanistically, in acute liver injury, free radicals generated by the metabolites of drugs or chemicals damage hepatocytes, leading to the release of damage-associated molecular patterns that induce inflammation and activate non-parenchymal cells, thereby initiating the regeneration process. Following acute liver injury, activated resident inflammatory cells, including KCs, and nonresident inflammatory cells from the bone marrow, are recruited to the damaged area to support regeneration. For example, activated KCs secrete IL-6, which facilitates the expression of several genes related to acute-phase proteins, cell-cycle regulation, redox balance, and anti-apoptotic mechanisms, thereby promoting the proliferation of remnant hepatocytes.^189^^–^^191^ Similarly, non-parenchymal cells such as HSCs and LSECs contribute to liver regeneration by inducing hepatocyte proliferation and sinusoidal remodeling through mutual signaling loops.^192^ LSECs are also activated by acute inflammation and produce HGF and Wnt2 to initiate liver regeneration. Following an acute injury, the increase in CXCR7 expression in LSECs works together with CXCR4 to activate the transcription factor Id1. This process releases pro-regenerative angiocrine factors and initiates regeneration.^193^ HSCs are transiently activated due to immune responses and adhere to hepatocytes during the initial stage of liver regeneration in rats,^194^ and increased ECM production will lead to an acceleration of the regeneration process. The activated HSCs secrete HGF, to initiate the liver regeneration while ECM acts as a scaffold for the proliferation of hepatocytes, provides mechanical stability in the damaged area, and helps to restore the architecture of the injured tissue.^34^

### Liver regeneration after chronic liver injury

**FIGURE 2:**
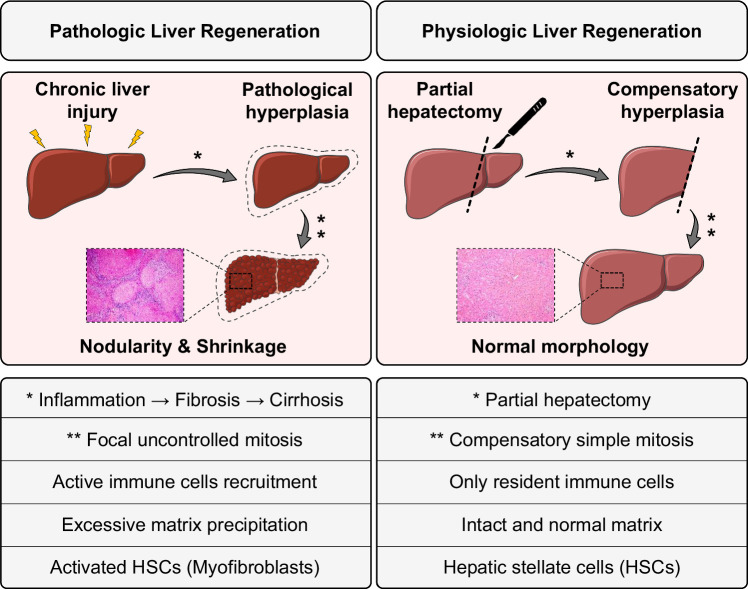
Different conditions that cause liver injury lead to different consequences in liver regeneration.

Chronic exposure to hepatotoxic agents, including xenobiotics, viral infections, and medications, leads to chronic inflammation and tissue remodeling, activation of HSCs, and stimulation of fibrogenesis, causing an imbalance between ECM synthesis and biodegradation.^195^ In chronic liver inflammation, the loss of hepatocytes continues alongside their proliferation.^191^ Long-term proliferation of hepatocytes is associated with fibrosis and HSCs activation, resulting in nodular hyperplasia^196^ and potentially HCC.^197^ Indeed, the morphology of the liver would change during this pathological regeneration, manifesting as nodularity and shrinkage of the hepatic tissue, which is different from physiological liver regeneration, characterized by compensatory hyperplasia and without any nodularity^198^ (Figure 2). Besides, damaged hepatocytes and activated HSCs release different cytokines and chemokines, which attract immune cells (chemotaxis) of liver tissue and infiltration of peripheral blood immune cells into the injury site.

Liver regeneration following chronic injury is a complex process that involves several cell types within the liver. Specifically, LPCs, cholangiocytes, and HSCs are considered key contributors to hepatocyte formation during regeneration. These cells interact with resident immune cells in various ways to facilitate the regenerative process.^199^ Studies have demonstrated the role of different cell lineages in the liver, which offer avenues to drive liver regeneration. For example, SOX9^+^ hepatocytes are considered bipotent progenitors, serving as the primary source of both hepatocytes and ductal cells during the regeneration process following CCl_4_-induced liver injury.^200^ Recently Peng et al^201^ reported a rare Gli1+ (glioma-associated oncogene 1)/epithelial cell adhesion molecule positive progenitor cell population characterized by both epithelial and mesenchymal identities contributing to liver repair and liver regeneration during chronic injury. Forkhead box protein A3 (FOXA3), a transcription factor, has been predominantly expressed in hepatocytes and cholangiocytes during the regeneration after PH and CCl_4_-induced liver injury in mice. FOXA3 ablation in mice caused exacerbation of CCl_4_-induced liver injury and delay in the regeneration process.^202^ This study further suggests that FOXA3 promotes liver regeneration by regulating the transcription of CCAAT/enhancer-binding protein beta (Cebpb). It has also been revealed that the levels of Cebpb and Ki67 were positively correlated with Foxa3 expression in livers in patients with chronic liver diseases.^202^

From the HSC perspective, during chemically-induced chronic liver injury, myofibroblasts produce excessive fibrous scar tissue, leading to hepatic fibrosis. Experimental studies have shown that when the underlying etiological agent is removed, liver regeneration begins, and the fibrosis remarkably regresses with the disappearance of myofibroblasts. A significant population of myofibroblasts undergoes apoptosis. Kisseleva et al^203^ demonstrated that approximately half of the myofibroblasts evade apoptosis and transform into a new phenotype, termed “inactive hepatic stellate cells,” which is similar to but distinct from the original quiescent HSCs during the recovery phase after CCl_4_ and ethanol intoxication. These inactive hepatic stellate cells exhibit decreased expression of fibrogenic genes such as collagen-α1(I), collagen-α1(II), α-SMA, TGFβRI, and TIMP1, while upregulating certain quiescence-associated genes (PPARγ and Bambi) to levels comparable to quiescent HSCs. The inactivation of HSCs has been implicated in the upregulation of anti-apoptotic genes, including Heat Shock Protein Family A 1a/b, which contribute to the survival of HSCs both in vitro and in vivo. HSCs have been recognized as a potential source of LPCs and may contribute to the formation of hepatocytes in the adult liver.^204^^,^^205^

SRY-related HMG box transcription factor 9^+^ LPCs have also been suggested to be responsible for one of the main sources of hepatocyte turnover in CCl_4_-intoxicated mice.^206^ LPCs act as regulatory or “dimmer switch” cells during chronic liver injury and regeneration. LPCs are usually dormant in the periportal region in the healthy liver; however, after chronic liver injury, they actively proliferate and yield transit-amplifying cells (or oval cells) called the ductular reaction (DR) in humans or oval cell proliferation in rodents.^207^^–^^209^ LPCs are significantly contributing to liver regeneration after partial hepatectomy or CCl_4_-induced liver injury.^206^ Growth differentiation factor 11 (GDF11) has been shown to play a critical role in the crosstalk between HSCs and LPCs during liver regeneration. Low levels of GDF11 have been associated with enhanced pro-fibrogenic factors, which activate HSCs to become fibrogenic and induce fibrosis via LPCs. During regeneration following chronic liver injury, the perisinusoidal microenvironment becomes less fibrogenic, with elevated levels of GDF11 promoting the expansion of LPCs and the recruitment of scar-resolving immune subsets.^210^ Large number of LPCs and biliary cell-derived yellow fluorescent protein^+^/hepatocyte nuclear factor 4α^+^ hepatocytes have been reported during regeneration after CCl_4_-induced liver injury in mice. The presence of such cells is correlated with the lower density of ECM, laminin, and/or other CTGF-dependent profibrotic signals that stimulate hepatocyte differentiation.^209^ Further, coculturing LPCs with activated HSCs isolated from mice intoxicated with thioacetamide for 6 weeks has been reported to reduce the fibrogenic potential of these HSCs within the fibrotic niche.^211^ Yap protein becomes activated in response to hepatocyte damage caused by ethanol and CCl_4_. Once activated, Yap forms complexes with TEAD transcription factors in the nucleus, driving transcriptional reprogramming that promotes hepatocyte proliferation and activation of progenitor cells.^212^ Based on experimental findings, Qian and colleagues have proposed a mechanism of activation, expansion, and differentiation of liver progenitor-like cells into hepatocytes during liver regeneration. After chronic liver injuries, dying hepatocytes express hedgehog ligands, which are shown to directly induce liver progenitor-like cells mediated regeneration and indirectly induce their proliliferation by activating HSCs into ECM producing phenotype.^213^ The lymphotoxin β receptor on HSCs, activated through the HIPPO/YAP pathway, could play a role in paracrine communication with neighboring LTβ-expressing liver progenitor-like cells. After activation, TWEAK, probably from recruited macrophage,^214^ exerts a selective mitogenic effect on liver progenitor-like cells by promoting their proliferation through its receptor, FGF-inducible 14.^215^ FGF-7 is involved in the proliferation of liver progenitor-like cells, while FGF-9 plays a role in the differentiation of progenitor-like cells into hepatocytes. Hepatic growth factor/c-met signaling,^216^ Wnt/β-catenin signaling,^217^ and thyroid hormone signaling^218^ have been found to be implicated in the differentiation of progenitor-like cells into mature hepatocytes.

Cholangiocytes transdifferentiate into hepatocytes through the mechanism of DR, which occurs in chronic liver injury. There is enough evidence suggesting that chronic liver injury causes DR-cells to differentiate into stress-resistant hepatocytes that replenish the liver. This process may be the cause of parenchymal rebuilding found in the livers of individuals with advanced-stage hepatitis.^219^ Actually, long-lasting liver injury is linked to an extensive DR and a decline in native hepatocyte regenerative capacity at a time-dependent level.^220^

Interestingly, Wnt/β catenin and Notch signaling play pivotal roles during liver regeneration. In fact, these pathways coordinate the biliary epithelial cell (BEC)–transitional liver progenitor cell (TLPC) hepatocyte transdifferentiating process. These findings indicate that Notch signaling inhibitors can enhance BEC to TLPC conversion. Moreover, Wnt/β catenin activation increases TLPC to hepatocyte conversion. Such mechanistic evidence may offer a potential therapeutic target for chronic liver injuries.^221^

Macrophages and NK cells play pivotal roles in liver regeneration. During liver regeneration, LPCs induce the recruitment of infiltrating macrophages to the injured liver through the CCL2/CCR2 and CX3CL1/CX3CR1 chemokine axes.^213^ Anti-inflammatory M2 macrophages promote tissue repair.^222^ Macrophages influence NK cells through the secretion of cytokines and complex cellular interactions. NK cells are also involved in LPC proliferation and MSC migration and recruitment towards immune cell populations via the secretion of CXCL7, thereby promoting regeneration.^223^ Innate immune cells, like monocytes, are also critically involved in liver regeneration. Classical monocytes express high levels of Ly6c, which are reportedly associated with promoting liver injury. Non-classical Ly-6Clow monocytes have been shown to be involved in tissue repair, inflammation resolution, and regeneration.^224^ Following mild liver injury, there is a phenotypic switch from classical to non-classical monocytes to accelerate tissue repair. However, during progressive liver injury, persistent inflammation leads to a failure in this phenotypic switch, impairing the repair process.^225^ Monocyte-derived CD11b^+^ macrophages, which are known to activate HSCs and promote fibrosis, are increased during thioacetamide-induced chronic liver injury.^226^ However, their population decreases during fibrosis resolution, contributing to a less fibrotic environment after thioacetamide-induced chronic liver injury.^211^ Similarly, in a CCl_4_-induced regeneration in mice, activation of D prostanoid receptor-1 enhanced Wnt2 transcription in resident KCs via a protein kinase A/CRE-binding protein-dependent pathway and facilitated hepatocyte proliferation through Frizzled8/β-catenin signaling,^227^ suggesting the crosstalk between KCs and hepatocytes through Wnt2 signaling during liver regeneration after chronic liver injury.

Altogether, liver regeneration after chronic liver injury is a multifaceted complex process that involves various cells, all interacting through many intricate signaling pathways. This complexity is further compounded by other factors such as the nature and the extent of the injury. All This makes the understanding of liver regeneration post chronic liver injury very challenging.

## CELLULAR AND MOLECULAR MECHANISMS

Several cytokines are responsible for the pathological regeneration of the liver, including TGF-β, which promotes the deposition of type I and III collagens in ECM,^228^^,^^229^ IL-1 β and TNF-α, which support the survival of HSCs,^230^ and IL-33, which indirectly activates HSCs through the STAT-6 pathway.^231^ Moreover, immune cells unintentionally play a determining role in the pathogenesis of liver regeneration. Regulatory T-cells (Tregs) and HSCs have a mutual crosstalk in an IL-2-dependent pattern. HSCs specifically stimulate Tregs, while Tregs have an opposite effect on HSCs; Tregs protect HSCs from natural killer (NK) cells' attack, and also have an inhibitory effect on activated HSCs.^232^ Activated NK cells release interferon-gamma (IFN-γ) through the JAK–STAT pathway to tackle hepatic fibrosis by destroying the activated HSCs. However, with sustained HSC activation during chronic liver injury, HSCs suppress NK cell activity and reduce their anti-fibrotic action. This is related to the increased metabolism of vitamin A, which produces retinoic acid and retinol metabolites that inhibit the activation of IFN-γ through the STAT-1 pathway by secreting suppressor of cytokine signaling 1 (SOCS1).^233^ Additionally, normal hepatocytes are heterogeneous in ploidy level, from 2n to 8n, which is a characteristic factor for liver homeostasis.^234^ After physiological/normal liver regeneration, the ploidy would be stored in hepatocytes; however, in chronic liver injuries, the ploidy level can be decreased.^235^ DNA damage might occur in both normal and during acute injury, which can be compensated by polyploidy and excessive expression of damaged alleles. However, in chronic liver injury, chromosome abnormality, especially in growth control genes (eg, EGF), can initiate neoplasia^236^ (Figure 1).

Another concept in pathological liver regeneration is associated with the role of hepatic resident stem cells. In chronic liver injury, the capacity of hepatocytes to maintain liver hemostasis may not be sufficient; therefore, a complementary mechanism could be activated to accelerate liver regeneration. In this regard, small bipotential resident stem cells, known as LPCs in humans or oval cells in rodents, could be activated to support the regeneration process.^237^ LPCs can differentiate into hepatocytes and biliary cells and, accordingly, play a crucial role in liver repair during constant hepatic or biliary damages such as viral hepatitis or biliary cirrhosis.^238^ However, the activation of LPCs may be associated with myofibroblasts activation, an alarming sight of liver regeneration in chronic damage settings.^239^ This issue is related to the microenvironment of LPCs, which contains ECM, growth factors, and cytokines.^240^ CTGF, a member of LPCs’ niche, releases heparin-binding proteins (HBP) and plays an essential role in liver fibrosis.^241^ CTGF, whose levels are increased in chronic liver damage, will exacerbate fibrogenesis by activating HSCs.^242^

MKK4 is essential for cell proliferation, differentiation, and apoptosis, as it modulates the JNK and p38 MAPK signaling pathways.^243^ Recent studies have focused on its inhibitors as potential therapeutics for liver diseases and cancer. Development of HRX215, a small molecule inhibitor of MKK4 kinase, has shown significant potential in promoting liver regeneration and preventing liver failure in preclinical studies.^244^ This small molecule was well-tolerated at all doses, with no significant changes in clinical or laboratory parameters. Notably, HRX215 reduced tumor progression in a diet-induced obese mouse model with NASH and advanced fibrosis.^245^^,^^246^ A phase I clinical trial showed favorable safety and pharmacokinetic properties for HRX215, supporting its further clinical investigation.^245^

## CONCLUSIONS

**FIGURE 3:**
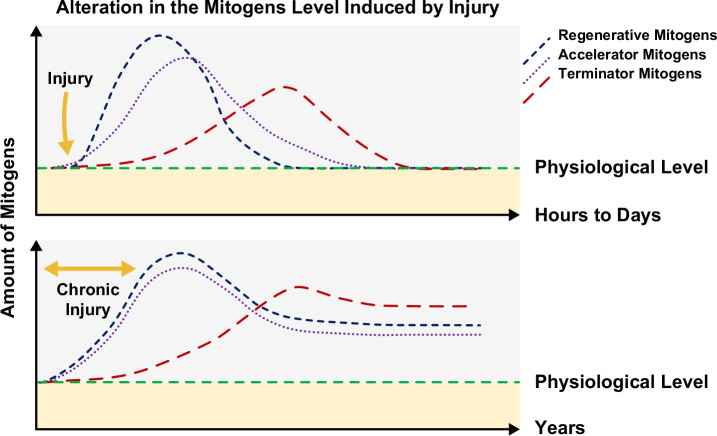
Alterations in mitogen levels lead to different outcomes. The start point, duration, and concentrations of mitogens are the main determinators of the liver regeneration’s fate.

Liver regeneration is an intricate process, implying various molecular and cellular mechanisms. At first glance, it may seem that the same mitogens are involved in promoting both normal and pathological liver regeneration. However, the most important difference between these 2 pathways, which lead to different fates, is the start time, duration, and different concentrations of regenerative, accelerator, and terminator mitogens (Figure 3). In contrast to pathological liver regeneration, which occurs after chronic liver damages, physiological liver regeneration typically takes place after acute injuries or liver resection. Pathological liver regeneration typically results in nodularity and shrinkage, whereas physiological liver regeneration is associated with liver enlargement. Comprehending both the positive and potentially negative outcomes of regeneration mechanisms holds the potential to unlock novel therapeutic opportunities. The knowledge accumulated over the past few decades, particularly through examining liver regeneration in rodents, is expected to facilitate this attempt significantly. The ultimate goal of this area of research is to harness the profound understanding of hepatocellular plasticity and renewal, and translate it into the development of targeted therapeutics for a broad spectrum of human liver diseases. By comprehending the intricate mechanisms underlying the ability of liver cells to adapt and regenerate, researchers aim to identify novel therapeutic approaches that can effectively address conditions such as liver fibrosis, cirrhosis, hepatitis, and even liver cancer. By leveraging this knowledge, we envision the development of tailored treatments that can promote liver regeneration, inhibit disease progression, and restore normal liver function. These efforts strive to offer hope and improved outcomes for patients facing liver diseases by unraveling the potential of hepatocellular plasticity as a therapeutic avenue and driving innovation in the field of liver medicine.
